# Transforming waste into worth: *Procambarus clarkii* carapace as a high-performance biosorbent for methyl red dye

**DOI:** 10.1038/s41598-026-44037-y

**Published:** 2026-04-02

**Authors:** Rofaida. F. H. Darweesh, Abdelaal S. A. Ahmed, Remon M. Zaki, Aldoshy Mahdy

**Affiliations:** 1 Environment Science Department, Faculty of Sugar and Integrated Industries Technology, Assiut University, Assiut, 71516 Egypt; 2 Chemistry Department, Faculty of Science, Al-Azhar University, Assiut, 71524 Egypt; 3 Zoology Department, Faculty of Science, Al-Azhar University, Assiut, 71524 Egypt; 4 Department of Chemistry, Faculty of Science, Assiut University, Assuit, Egypt

**Keywords:** *Procambarus clarkii*, Anionic dye, Adsorption, Biosorbent, Water treatment, Chemistry, Ecology, Ecology, Environmental sciences, Materials science

## Abstract

Low-cost and sustainable biosorbents for the treatment of dye-containing wastewater are an area that needs to be addressed in the near future. In the present study, waste from the *Procambarus clarkii*, an invasive crayfish, was used as a potential biosorbent to remove methyl red dye from aqueous solutions. The waste was found to have a high adsorption capacity, with a maximum capacity of 14.39 mg/g under optimum conditions. The noteworthy aspect was that the biosorbent, with a relatively low surface area (19.13 m²/g), was able to remove 97% of the dye at pH 7 in 2 h, thus proving the importance of surface functional groups in the removal of the dye. The kinetics revealed that the adsorption followed a pseudo-second-order equation. The thermodynamic parameters revealed that the adsorption was a spontaneous and exothermic process. Regeneration experiments revealed that the *Procambarus clarkii* biosorbent retained about 70% and 50% of the original adsorption capacity after the second and third cycles, respectively. In conclusion, this study has shown that the carapace of P. clarkii is an environmentally friendly and economically viable biosorbent, which has the dual advantage of wastewater treatment and biological waste utilization.

**Supplementary Information:**

The online version contains supplementary material available at 10.1038/s41598-026-44037-y.

## Introduction

Water pollution caused by both organic as well as inorganic contaminants is one of the most serious environmental concerns faced by the world today, mainly because of untreated or poorly treated industrial waste discharges. Among these pollutants, heavy metals as well as synthetic dyes are of serious concern because of their long-lasting effects on both the environment as well as human health^1^. These pollutants have the capacity to penetrate aquatic food chains, thus creating long-lasting health as well as environmental concerns. Among these pollutants, synthetic dyes have also raised serious concerns because of their high toxicity, carcinogenicity, as well as their high resistance to biodegradation. Because of their chemical stability as well as aromatic structure, synthetic dyes are highly resistant to biodegradation; hence, removal of synthetic dyes from industrial waste is a serious environmental concern^2,3^.

It is estimated that about 7 × 10⁴ tons of toxic dyes are discharged into wastewater streams every year from textile, paper, and leather industries.³ In addition, other organic pollutants such as phenols, oils, pesticides, and polycyclic aromatic hydrocarbons are also contributing to the problem^4,5^. Amongst the dyes, methyl red (MR), a widely used anionic azo dye with a polyaromatic structure, is a major pollutant that is non-biodegradable. The toxicity, mutagenicity, and stability of MR make it a major pollutant that requires immediate attention for its removal. The toxicity of MR is a major cause of severe adverse effects on the skin, eyes, and respiratory system^6^. To meet increasingly stringent environmental quality standards, industrial effluents must undergo advanced treatment to eliminate these recalcitrant compounds^7^.

Although the World Health Organization (WHO) does not indicate the specific permissible level of individual synthetic dyes, such as methyl red, in wastewater, it does emphasize the importance of the treatment of industrial wastewaters to prevent the entry of harmful organic pollutants into water resources in the environment due to their toxic, mutagenic, and carcinogenic effects. Generally, the environmental regulations indicate effluent quality standards for conventional water quality parameters, and not specific concentration levels of individual dyes, since synthetic dyes are often regulated indirectly through criteria for water quality rather than specific criteria for dyes^8^. For instance, in characterization studies of textile wastewaters, the dye concentrations in the effluent are often reported in the range of 10–250 mg/L, corresponding to different concentrations of reactive dyes^9^.

Several technologies of treatments have been developed to desorb polluting materials from industrial effluents, including chemical precipitation, ion exchange, electrodeposition, membrane separation, and adsorption^10^. Among these, chemical precipitation has traditionally been regarded as a low-cost and simple process; however, its limited removal efficiency and formation of secondary sludge restrict its application on large scales^11^. More advanced technologies, such as ion exchange and reverse osmosis, are marked by high efficiencies of removal but are often constrained by high operational and maintenance costs, making them less viable for industrial-scale use^11–13^. Compared to them, adsorption particularly employing low-cost, naturally occurring sorbents is one of the most cost-effective and environmentally sound methods for the removal of toxic metal ions and dyes from liquid phases. Previous studies revealed the high efficacy of adsorbents such as granular activated carbon and chitin in desorption of various contaminants from wastewater^14^.

Recently, there has been interest in biopolymer-derived adsorbents owing to their biodegradability, abundance in nature, and ability to target pollutants. Of these, chitin and its deacetylated form, chitosan, are well-known. However, traditional methods of preparing chitin and chitosan involve several chemical processes, such as demineralization and deproteinization, which are expensive and environmentally hazardous. To make the process easier and more environmentally friendly, the use of crustacean carapaces as biosorbents has been suggested.

In this research, we concentrate on the *Procambarus clarkii* species, a freshwater decapod crayfish, which has turned into an aggressively reproducing invasive species in the Nile River ecosystem in Egypt. This species is harmful to the ecosystem and environment, competing with native biodiversity, fish egg consumption, and habitat dynamics, thus decreasing fish populations. On the other hand, its carapace is a byproduct of seafood, consisting of 40% calcium carbonate, 30% protein, and 30% chitin, which has favorable physicochemical properties for adsorption. Previous research has shown that biowaste from crustaceans can outperform some natural and synthetic adsorbents in heavy metal and organic pollutant removal^15^. The outstanding adsorption efficiency of this material can be attributed to the hierarchical, mineralized structure of the carapace, which consists of a chitin-protein organic matrix and calcium carbonate^16,17^.

Several studies have also been conducted to explore the biosorbent potential of crustacean shell types, including crab, shrimp, and lobster shell exoskeletons for dye and heavy metal biosorption. The choice of the model pollutant was based on the persistence of the compound in the environment, its high toxicity, and its presence in industrial effluents. In this work, we propose a new approach to valorize the invasive freshwater crustacean *Procambarus clarkii* as a biosorbent for wastewater treatment at low cost in its raw form.This work shows that the hierarchical microstructure and the chemical functionality of the raw *Procambarus clarkii* carapace are crucial factors in achieving high adsorption efficiency, reaching 97%, without the need to chemically modify the adsorbent. In contrast to other activated or chemically modified adsorbents used for wastewater treatment, our biosorbent does not require any pre-treatment but presents high adsorption efficiency because of its hierarchical structure and high content of functional groups. This approach not only presents a reliable solution for dye removal from wastewater but also contributes to ecosystem management by tackling an ecologically harmful species.

## Experimental section

### Materials

All chemicals used in this study were of analytical grade. Methyl red (C_15_H_15_N_3_O_2_), distilled water (H_2_O), hydrochloric acid (HCl, 37%), and sodium hydroxide (NaOH, > 98%) were from Alpha Chemika, India. All used reagents were of analytical purity.

### Preparation of *P. clarkii* carapace

**Fig. 1:**
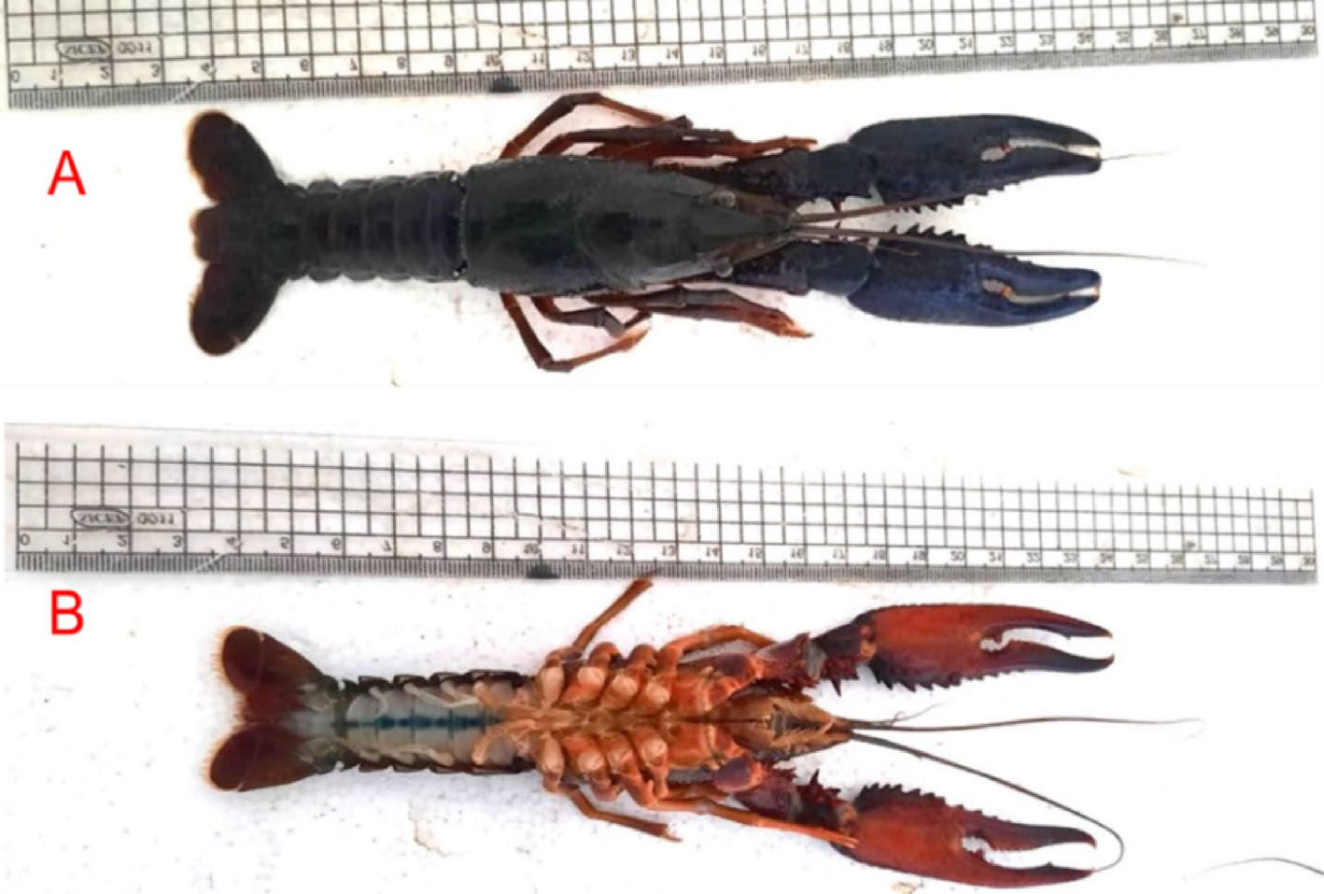
Optical image of *Procambarus clarkii* (**a**) dorsal view (**b**) ventral view.

*Procambarus clarkii* (red swamp crayfish) samples were collected from the Nile River along Assiut Governorate, Egypt (Fig. 1A, B). The length of the specimens averaged 20.3, 19.7, and 18.6 cm, and the corresponding weights averaged 29.2, 28.7, and 28.7 g. For examination of the outer morphology and preparation of the carapace-based adsorption material, the crayfish samples were first cleaned well under running water to remove surface contaminants. The organisms were cut open, and the carapaces separated gently and placed in a beaker containing boiling water. The samples were then heated at 2 h on a magnetic stirrer to expel any sticking organic residues. The carapaces were oven-dried at 90 °C for 3 h under any chemical reagent. After cooling to room temperature, the dried carapaces were ground into fine powder for the purpose of obtaining the microstructured biosorbent material, which was then used in the adsorption experiments.

### Characterization

The carapace of *Procambarus clarkii* was characterized using a variety of analytical techniques. Fourier Transform Infrared Spectroscopy (FTIR) was employed to identify the functional groups present in the biosorbent, both before and after adsorption. To investigate the crystallinity of the prepared materials, X-ray Diffraction (XRD) analysis was performed using a Bruker D8 Advance diffractometer operating at 40 kV with a monochromatized Cu-Kα radiation source. The specific surface area and pore size distribution were analyzed via nitrogen adsorption–desorption isotherms using a Micromeritics ASAP 2020 HD88 instrument. The Brunauer–Emmett–Teller (BET) method was used to calculate surface area. Finally, the concentration of MR dye in solution was measured using a UV-Visible spectrophotometer (Nicolet Evolution 300, Cany Precision Instruments Co., Ltd., China) at a wavelength of 520 nm. The surface charge and size distribution of the prepared materials were detected using Zeta sizer nano ZS90, Malvern Instruments Ltd., U.K. at 25 C. Using a field emission scanning electron microscope (SEM, JSM-6610, JEOL, Japan), the sample morphologies were determined. The surface element composition and chemical valence state of composite aerogels before and after adsorption of MR were analyzed by X-ray photoelectron Spectrometer (XPS, Thermo Kalpha, USA). Transmission electron microscope (TEM) pictures were captured using a JEM-2100 F field emission microscope (JEOL Ltd., Japan) at an accelerating voltage of 200 kV.

### Adsorption study

The adsorption processes of MR were performed in a 40 mL beaker under a magnetic stirrer. The effects of solution pH, contact time, temperature, dye concentration, and adsorbent dosage on the dye removal percentage by the *Procambarus Clarkii* investigated by batch adsorption technique. In each experiment, 0.1 g of adsorbent material was mixed with 50 mL of dye solution at an initial 20 mg/L concentration. The pH of dye solutions was adjusted by 0.1 M HCl and 0.1 M NaOH aqueous solutions. The remaining concentration of dyes was measured using a UV–visible spectrophotometer at a wavelength of 520 nm for MR. Similar experimental procedures were also used to investigate the influence of the initial dye concentration (5 mg/L–50 mg/L), contact time (2–120 min), temperature (30 − 70)ºC, solution pH (3–12), and adsorption dose (0.01–0.10 g) on the adsorption process. The removal percentage (R %) and the adsorption capacity (q_e_; mg/g) of dye were determined by Eqs. 1& 2, respectively.

1 $$R{\text{ }}\left( \% \right) = \frac{{C0 - Ce}}{{C0}} \times 100$$

2 $$q_{e} = \frac{{\left( {C0 - Ce} \right)}}{M}~V$$

Where C_0_ and C_e_ are the initial and final dye concentration (mg/L), respectively, Vis the volume (L), and M is the mass of the adsorbent (g).

## Results and discussion

### Characterization of structure and morphology

**Fig. 2:**
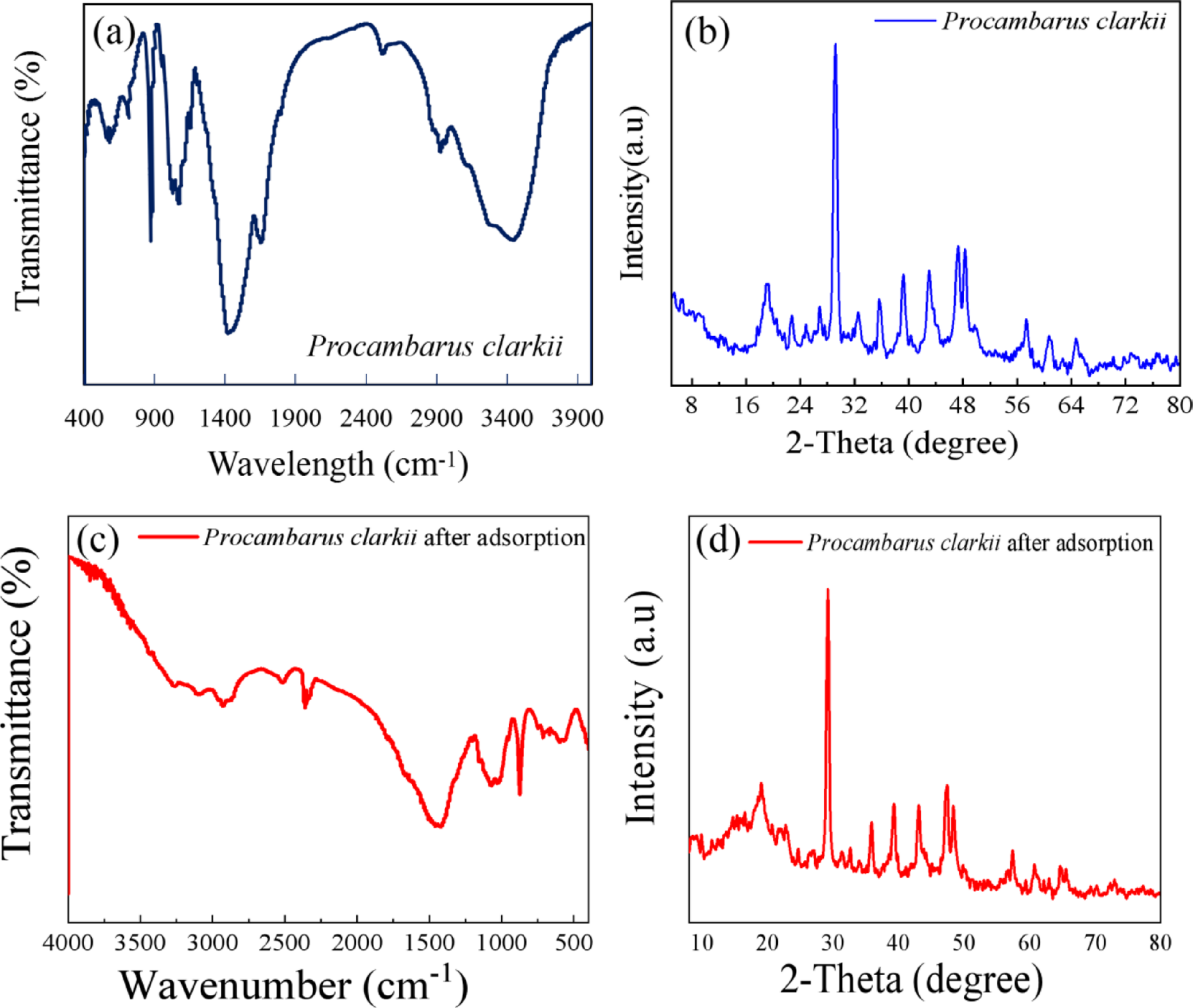
FT-IR spectra of *Procambarus clarkii* carapace before (**a**) and after MR dye adsorption (**c**). XRD patternn of *Procambarus clarkii* carapace before (**b**) and after MR dye adsorption (**d**).

The FT-IR spectrum of *Procambarus clarkii* carapace is shown in Fig. 2a. The band at 3435 cm^− 1^ is assigned to the -OH group. The bands at 2961 and 2926 cm^− 1^ represent symmetric stretching of the CH_2_ group. The bands at 1659 and 1535 cm^− 1^ can be assigned to the scissor bending of the C = O amidic group. The bands at 1154 and 1113 cm^− 1^ represent the C-N stretch of amide groups or the C-O stretch of the C-OH group^18^. The characteristic stretching vibration of the CO_3_^−2^ group can be observed at the bands 1416 and 873 cm^− 1^^19,20^.

The FT-IR spectra after the adsorption of MR (Fig. 2c) have these bands with a minor shifts in the bands, indicating the interactions between the MR molecules and the -NH2, -OH, and CO_3_^−2^ groups through electrostatic attraction, H-bonding, and ion exchange, respectively. The lack of the disappearance of the bands and the lack of the formation of new bands confirm that the adsorption process is not altering the chemical structure of the biosorbent material.

As shown in Fig. 2b, the XRD pattern reveals a series of distinct diffraction peaks corresponding to calcite, located at 20.04°, 29.40°, 36.00°, 39.40°, 44.30°, 47.80°, and 48.70°. These peaks confirm the crystalline nature of the carapace and indicate the presence of calcite as the primary mineral phase. The XRD patterns of the *Procambarus clarkii* carapace after the adsorption of MR (Fig. 2d) have the same characteristic diffraction peaks, which correspond to the calcite phase of CaCO_3_, indicating that the crystallinity of the carapace is unaltered after the adsorption process. The lack of new diffraction peaks and the unaltered positions of the diffraction peaks confirm that the adsorption of MR is mainly through surface interactions and not through any structural or mineralogical changes.

**Fig. 3:**
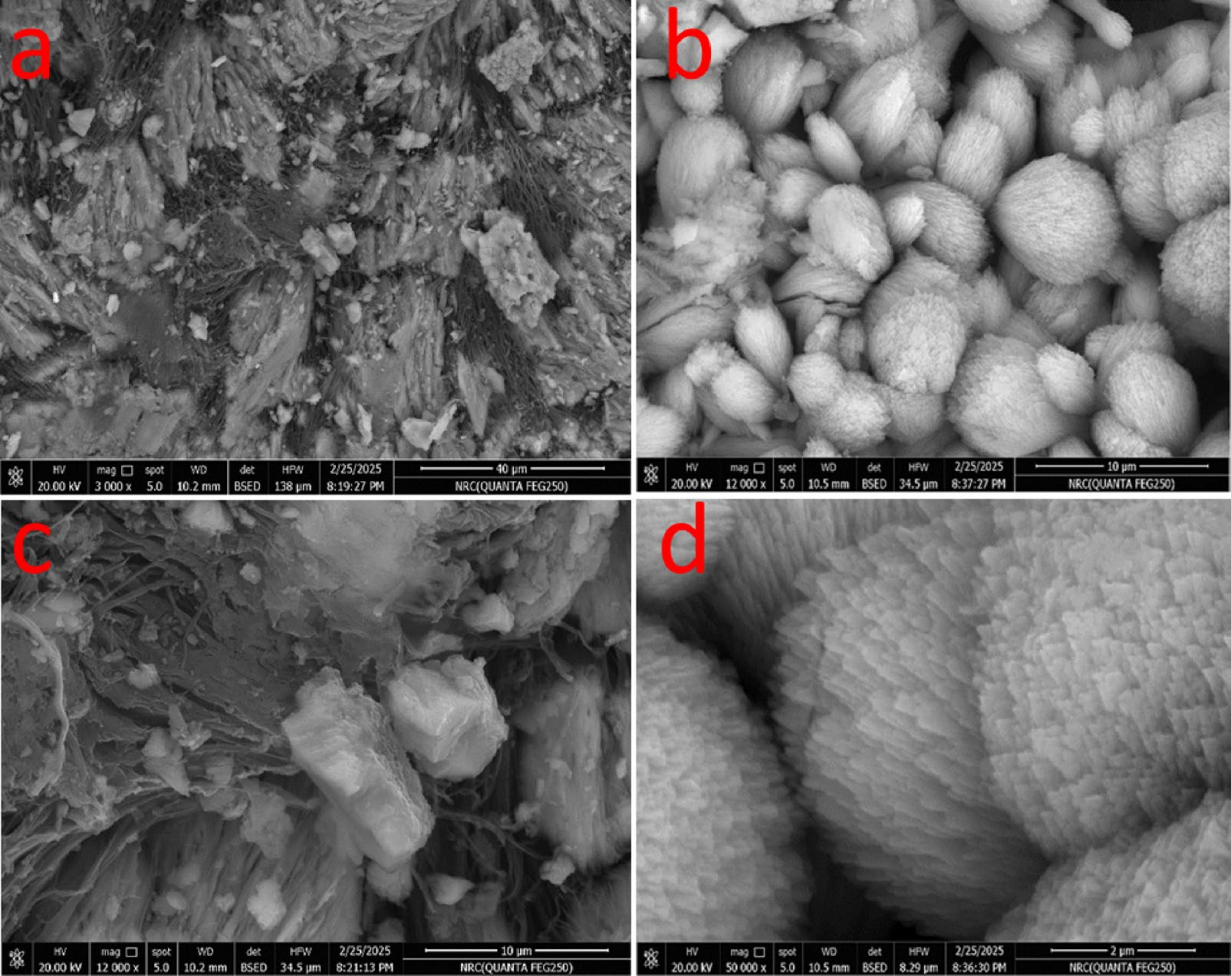
(**a**-**d**) SEM of *Procambarus clarkii* carapace at different magnifications.

Figure 3a, show hierarchical architecture of the biosorbent in respect of rough and heterogeneous surface, while Fig. 3b displayed aggregated spherical–flower-like particles, and lamellar layered morphology with broken sheets (Fig. 3c). On higher magnification (Fig. 3d), fibrillar bundles at the nanoscale are evident. This multi-scale structure contributed by the surface roughness, porous layers, and fibrillar nanostructures, contributes to the high surface area and abundant active sites and therefore the high adsorption capacity of the carapace material.

**Fig. 4:**
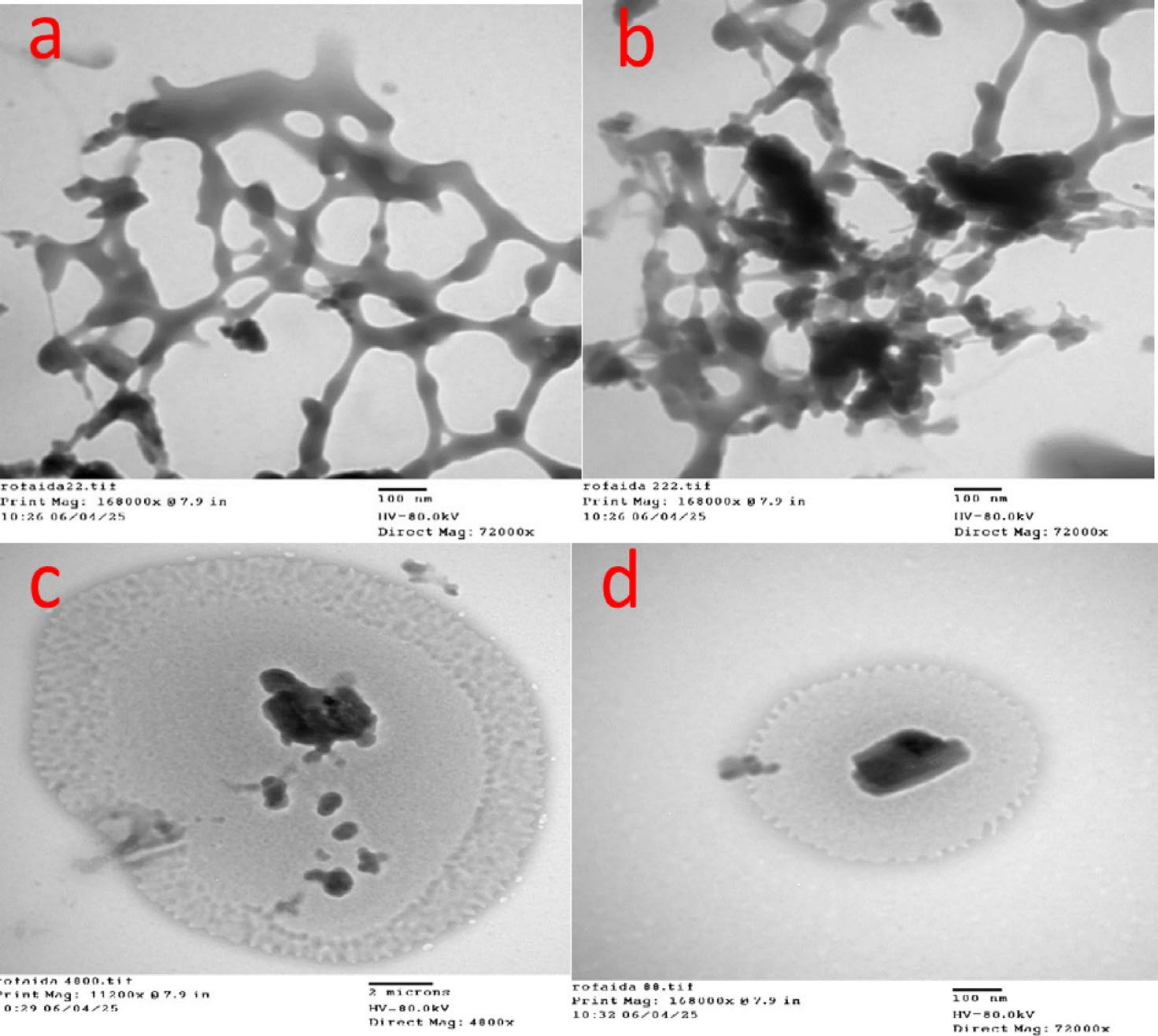
(**a**-**d**) TEM of *Procambarus clarkii* carapace.

The interior structure was described via TEM. Figure 4 shows the TEM images of the *Procambarus clarkii* carapace at different magnifications. Figure 4 (a, b) represents a sponge-like network structure, being porous at high magnification, thereby confirming the presence of nanoscale interconnected pores in the chitin–CaCO₃ matrix. Figure 4c shows a big particle with denser core and lighter shell, while Fig. 4d suggests core–shell morphology with mineral-rich center being covered by an organic chitinous layer. This porosity, core-shell nanostructure, and mineral-organic interaction hierarchical nanostructure enhances the surface area and offers abundant functional groups, thus adding to the high adsorption of the carapace.

**Fig. 5:**
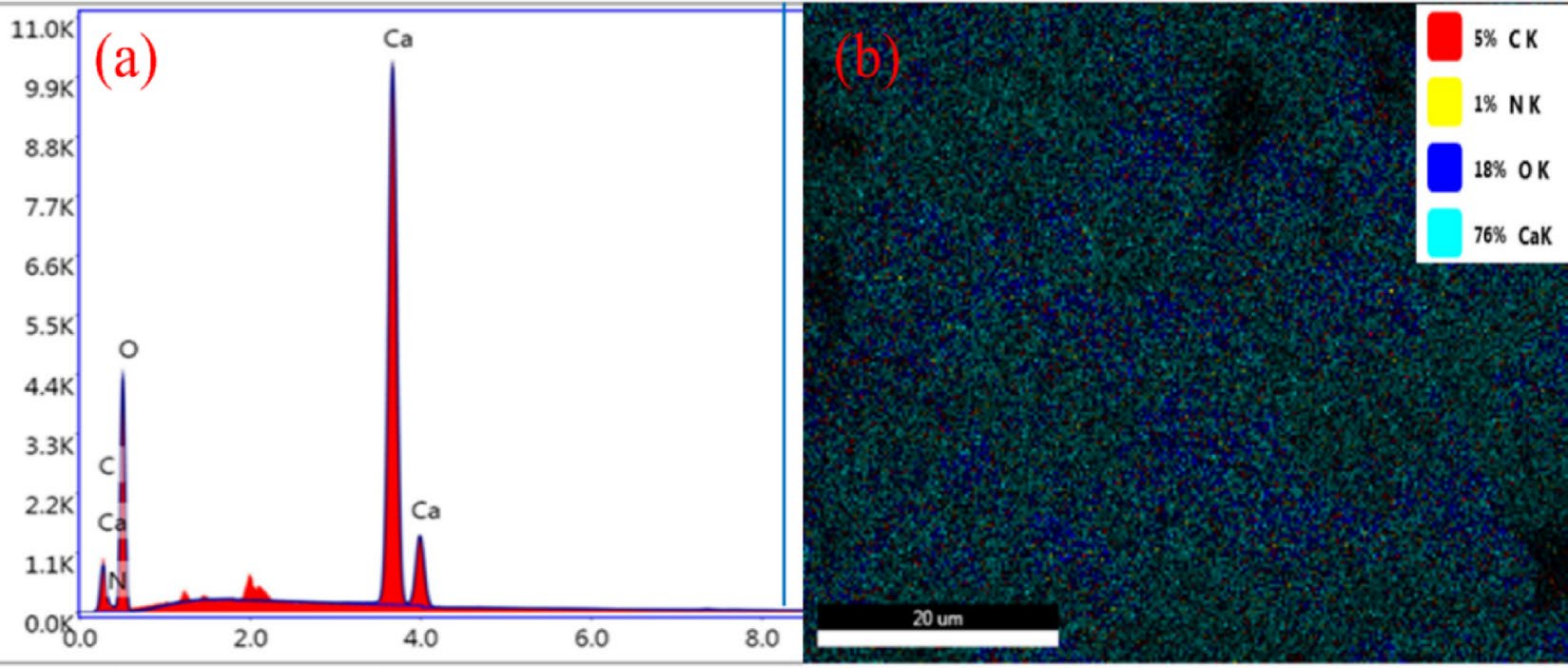
(**a**) EDX and (**b**) Element mapping of *Procambarus clarkii* carapace.

EDX spectrum of *Procambarus clarkii* carapace in Fig. 5a showing presence Ca, O, C, and N elements, indicating that there is a mineralized chitin-CaCO₃ matrix. Elemental mapping in Fig. 5b showing homogeneous distribution of Ca (cyan, 76%), O (blue, 18%), C (red, 5%), and N (yellow, 1%). Aqueous layering of calcium and oxygen confirms inorganic mineral structure, and addition of carbon and nitrogen is consistent with organic chitin–protein structure. The ratio confirms the hybrid organic–inorganic structure of the carapace, which confers structural stiffness and functional richness for adsorption.

**Fig. 6:**
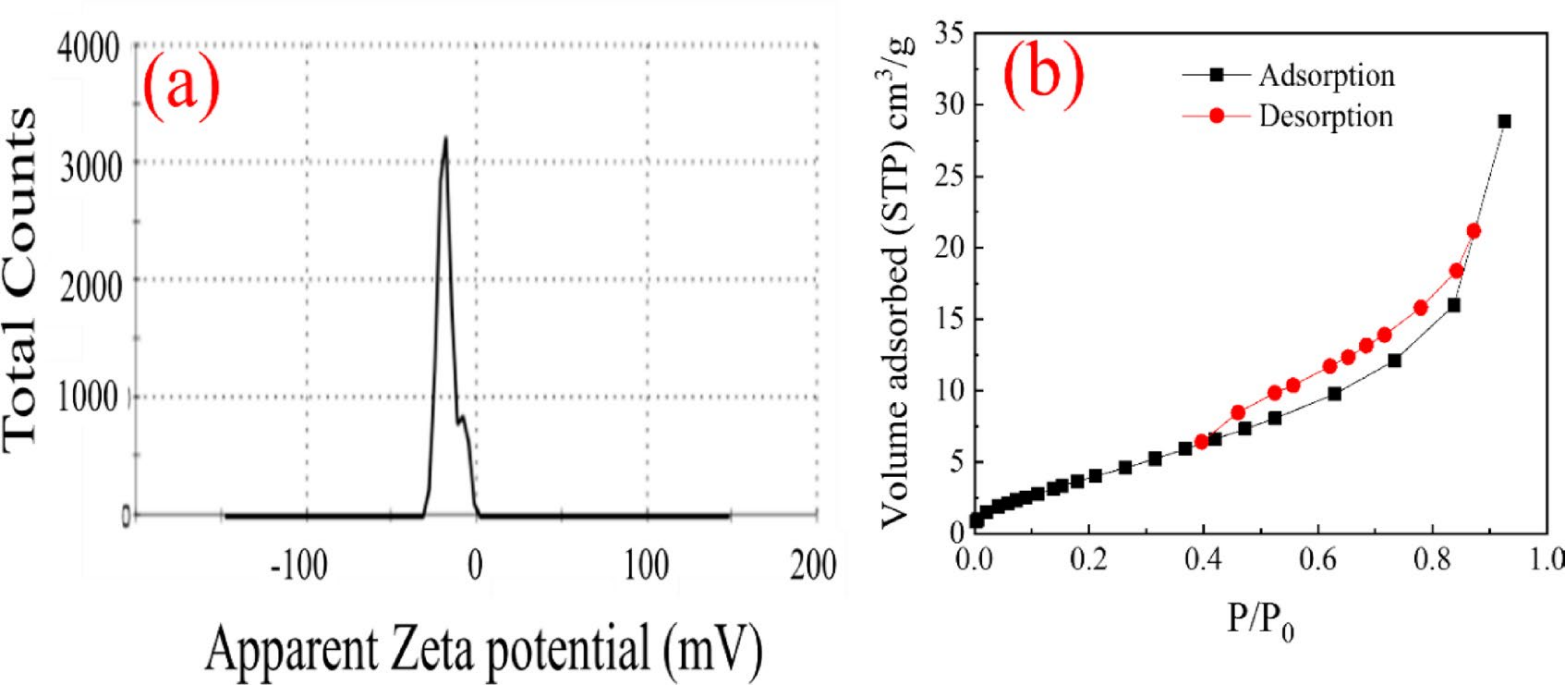
(**a**) Zeta potential and (**b**) nitrogen adsorption-desorption isotherm of *Procambarus clarkii* carapace.

Zeta potential analysis was also utilized to identify the point of zero charge (pHpzc) and assess its influence on MR adsorption. As depicted in Fig. 6a, Procambarus clarkii carapace has a positive charge at a pH near neutrality, with a zeta potential value of approximately + 17 mV. This indicates that the point of zero charge is above a pH near 7. At pH values lower than the point of zero charge, the carapace surface groups (-NH2, -OH) tend to be protonated and therefore have a positive charge, favoring electrostatic adsorption of negatively charged dye molecules.

MR is a type of azo dye with a predominantly anionic charge due to its deprotonated structure at pH near or above neutrality. Thus, the optimal adsorption efficiency at a pH value of 7 (89.9%) may be attributed to the strong electrostatic interaction between the positively charged biosorbent and negatively charged MR molecules. However, as the pH value increases above the point of zero charge, the carapace surface tends to be negatively charged due to the deprotonation of functional groups. This causes a repulsive force between the adsorbent and MR anions, resulting in a corresponding reduction in adsorption efficiency. At strongly acidic pHs (pH < 5), despite the high protonation state of the biosorbent surface, the partial protonation of the MR molecules and the presence of excess H+ ions may affect the dye-surface interaction. Therefore, the overall adsorption process over the entire pH range is controlled by the joint effect of changes in the surface charge compared to the pHpzc and the pH-dependent speciation of the methyl red.

The surface area is very important in the adsorption process. Nitrogen adsorption-desorption isotherms of crayfish carapace are presented in Fig. 6b. In spite of its rather low BET surface area (19.13 m^2^/g) for Procambarus clarkii carapace material, in comparison with other conventional high-surface-area adsorbents like activated carbon, this material demonstrates rather good adsorption efficiency. This observation is also in agreement with other observations in the literature that, in biosorption processes, surface chemistry and availability of surface functional groups may play a more important role than surface area. Reviews of low-cost adsorbents and biomass-based adsorbents emphasize that a wide range of raw biomaterials with low BET surface area may demonstrate rather good adsorption efficiency in dye or heavy metal removal^21^. These reviews emphasize that biomaterials with low BET surface area may be very effective in adsorption due to the availability of a high density of surface functional groups like hydroxyl, amino, and carboxyl groups, capable of specific adsorption mechanisms like electrostatic interaction, ion exchange, and complexation. The effectiveness of raw agricultural wastes in dye removal, despite low BET surface area, has been reported in several studies. The adsorption was attributed to a high density of adsorption sites rather than a high surface area^22^. This observation has also been emphasized in reviews on biosorption mechanisms^23^. As a result, the high functional group content, porosity, and favorable electrostatic properties of the P. clarkii carapace matrix contribute to the adsorption activity in a synergetic manner, making the P. clarkii matrix comparable to the aforementioned biosorbents in that the surface chemistry plays a significant role rather than the BET surface area.

### Adsorption study

#### Adsorption capacity and adsorption isotherm

**Fig. 7:**
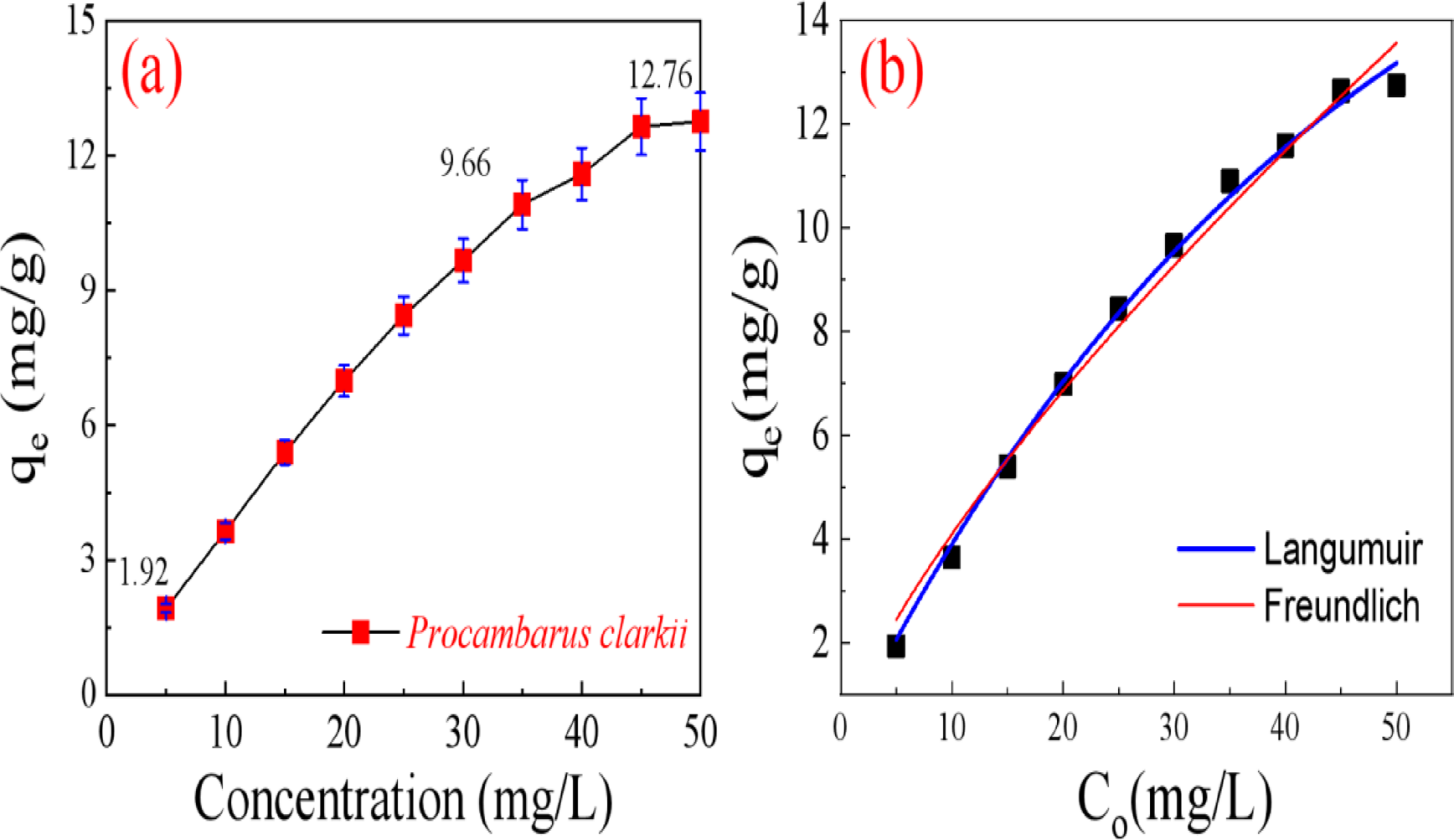
(**a**) Effect of MR dye initial concentration on adsorption capacity. (**b**) The experimental and adsorption isotherm.

The adsorption capacity of *Procambarus clarkii* was evaluated at various initial MR dye concentrations, as presented in Fig. 7a. In a typical test, 0.10 g of the adsorbent was added to 40 mL MR dye solutions with concentrations ranging from 5 to 50 mg/L. The pH of each solution was adjusted to 7 and the suspensions were shaken at 25 °C for 2 h. As observed, the adsorption capacity increased progressively with the initial dye concentration, demonstrating a sharp rise at lower concentrations, a gradual and progressively decreasing rise. Specifically, the adsorption capacity rose from 1.92 to 12.76 mg/g as the MR concentration increased from 5 to 50 mg/L. This is because increased electrostatic repulsion between surface adsorbed MR molecules and other molecules in the bulk solution at high concentrations prevents additional adsorption from taking place^24,25^.

In order to investigate the nature of the adsorption and the equilibrium behavior between the adsorbent-adsorbate molecules, two adsorption isotherm models (Langmuir and Freundlich) were applied to fit the experimental data^26^. As the linear models (See Supplimentry data; Fig.S1; Table S1) not well fit our experimental data, we utlise the non-linear fitting models. The non-linear plots are in Fig. 7b and the related parameters are in Table 1.

The Langmuir model assumes that the adsorbent surface is homogeneous with a finite number of identical sites; thus, a monolayer of adsorbate is chemically attached to the surface of the adsorbent. The non-linear form of the Langmuir isotherm can be expressed by Eq. 3^27^.

3 $$q_{e} \frac{{q_{{\max }} K_{L} C_{e} }}{{1 + K_{L} C_{e} }}~~~$$

Where C_e_ (mg/L) is the equilibrium concentration, qmax (mg/g) is the maximum adsorbation capacity, and K_L_ (L/mg) is the separation factor related to the adsorption affinity of the binding sites.

The Freundlich model is used to describe the heterogeneous surfaces, in which its non-linear form can be expressed by Eq. 4.

4 $$q_{e} = K_{f} C_{e}^{{1/n}}$$

Where Kf (L/g) is constant represents the adsorption capacity. The adsorption power (1/n) is represent the adsorption intensity and used to show the ideality and limit of the adsorbent/adsorbate interaction.

From the isotherm plots are shown on Fig. 7b and the estimated parameters are categorized in Table 1, the higher R^2^ value suggests that the Langmuir isotherm model best describes the experimental data. This implies that a surface with uniform coverage of localized adsorption sites has produced a dye monolayer.^28^ The interaction between an absorbent and an adsorbate is typically described by the Langmuir constant K_L_. The estimated K_L_ value for MR adsorption by *Procambarus clarkii* is 0.022 L/mg. The maximum adsorption capacities of MR onto Procambarus clarkii were estimated to be 19.75 mg/g.

**Table 1:** Parameters of non-linear Langmuir and Freundlich isotherm models for adsorption of MR on *Procambarus clarkii* carapace.

Adsorbent	Langmuir model			Freundlich model		
	K_L_ (L/mg)	q_m_ (mg/g)	*R* ^2^	K_f_ (mg/g)	*N*	*R* ^2^
*Procambarus clarkii*	0.02	19.75	0.996	0.75	0.74	0.985

#### Effect of pH

**Fig. 8:**
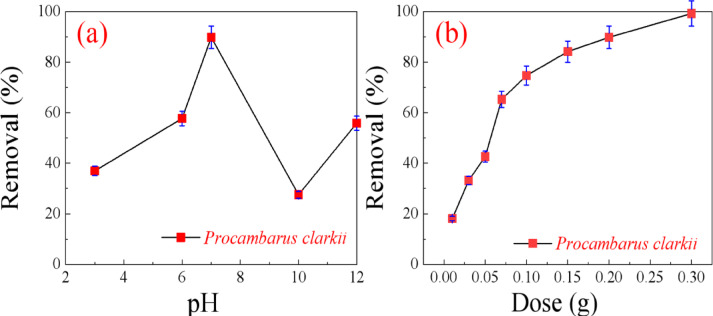
(**a**) Effect of pH and (**b**) adsorbent dose on MR dye removal.

One of the most significant parameters for dye adsorption is the pH of the solution since it regulates both the surface charge of the adsorbent and the ionization of the dye molecules. To establish the optimal pH for MR removal by *Procambarus clarkii*, batch adsorption experiments were carried out at a pH level of 3–12. In all experiments, 0.10 g of biosorbent was put into contact with 40 mL of MR dye solution (25 mg/L), and the pH was adjusted using 0.1 M HCl or 0.1 M NaOH. The suspensions were magnetically stirred at 25 °C for 120 min. As indicated in Fig. 8a, the adsorption efficiency dropped step by step with the rise of solution pH. The maximum adsorption efficiency of 89.9% appeared at pH 7, indicating that the neutral environment is most favorable for the adsorption of MR dye on *Procambarus clarkii*.

#### Effect of amount of adsorbent

Adsorbent dosage is a critical parameter to optimize adsorption efficiency and minimize material wastage once equilibrium is attained. For the current research, different adsorbent doses ranging from 0.01 to 0.10 g were individually mixed with 25 mL of MR dye solution of 25 mg/L at pH 7. The mixes were stirred at room temperature for 2 h. As indicated in Fig. 8b, the percentage of MR dye removal increased consistently with a higher adsorbent dose from 0.01 to 0.30 g. This increase can be explained by the higher number of available active sites on the surface of the adsorbent for effective interaction with dye molecules^29,30^.

#### Effect of contact time and adsorption kinetics

**Fig. 9:**
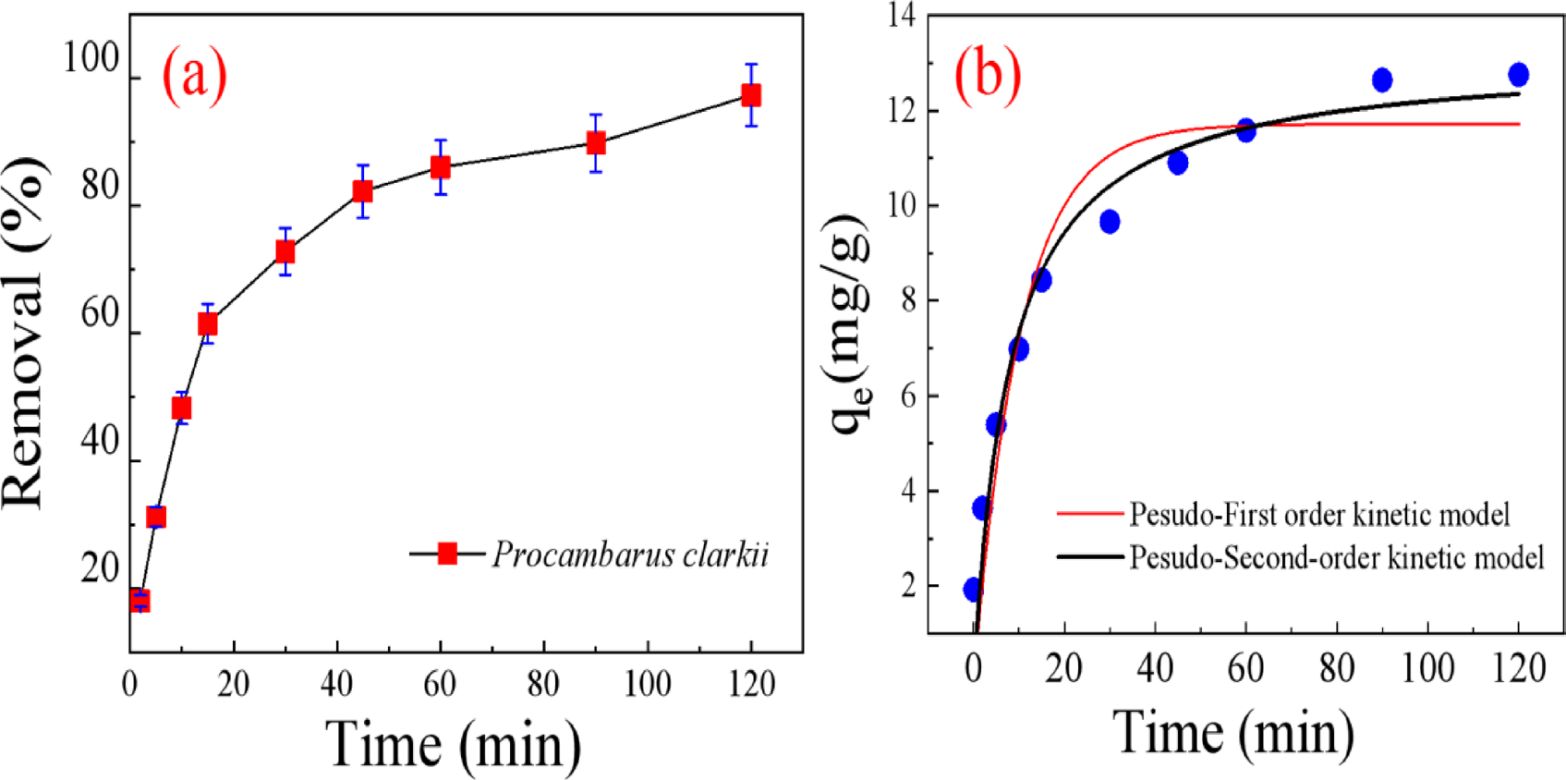
Effect of (**a**) contact time and (**b**) non-linear pseudo-first and pseudo-second order kinetic models for adsorption of MR on *Procambarus clarkii*.

The interaction time of the adsorbent with the adsorbate is an important parameter in the optimization of adsorption-treated wastewater. The effect of contact time on the effectiveness of removal of MR dye by *Procambarus clarkii* was investigated in a series of experiments using solutions of the dye with an initial concentration of 25 mg/L at pH 7. The experiments consisted of the addition of 0.10 g of adsorbent to 40 mL of dye solution and shaking for varying contact times between 2 and 120 min at 25 °C. As presented in Fig. 9a, removal efficiency increased with contact time to 97.3% after 120 min. The initial fast adsorption is due to the large number of active sites on the surface of the adsorbent^31^. With continued adsorption, the sites gradually become saturated with the dye, which leads to the plateau that indicates equilibrium where further uptake of the dye was restrained by repulsive intermolecular forces between adsorbed and un-adsorbed molecules in solution.

Adsorption kinetic experiments were conducted to get a better understanding of the mechanism and adsorption rate. As the linear models (See Supplimentry data; Fig.S2; Table S2) not well fit our experimental data, we utlise the non-linear fitting models. Two linear kinetic models, the pseudo-first-order model and the pseudo-second-order model, were employed to describe the adsorption behavior of MR dye onto *Procambarus clarkii*. The pseudo-first order model is expressed by Eq. 5.^32^

5 $$q_{t} = q_{e} \left( {1 - e^{{ - k_{1} t~}} } \right)~$$

Where qe and qt are the adsorbed dye (mg/g) at equilibrium and at time t (min), respectively. k_1_ is the rate constant (min^− 1^).

The pseudo-second-order model is expressed by Eq. 6.

6 $$q_{t} = \frac{{q_{e}^{2} k_{2} t}}{{1 + k_{2} q_{e} t}}~$$

Where k_2_ is the rate constant (g/mg.min).

From the fitted plots are represented in Fig. 9b, and their related parameters are in Table 2, the adsorption of MR dye onto *Procambarus clarkii* obey the pseudo-second-order kinetic model, as evidenced by R² values. Furthermore, the observed q_e_ values calculated using the pseudo-second-order kinetic mode are close to the experimental values.

**Table 2:** Parameters of the pseudo-first and pseudo-second order kinetic models for adsorption of MR on *Procambarus clarkii*.

Adsorbent	Pseudo first order			Pseudo second order		
	q_e_ (mg/g)	K_1_ (1/min)	*R* ^2^	q_e_ (mg/g)	K_2_	*R* ^2^
*Procambarus clarkii*	11.72	0.096	0.899	13.17	0.0096	0.948

#### Effect of temperature and thermodynamic analysis

**Fig. 10:**
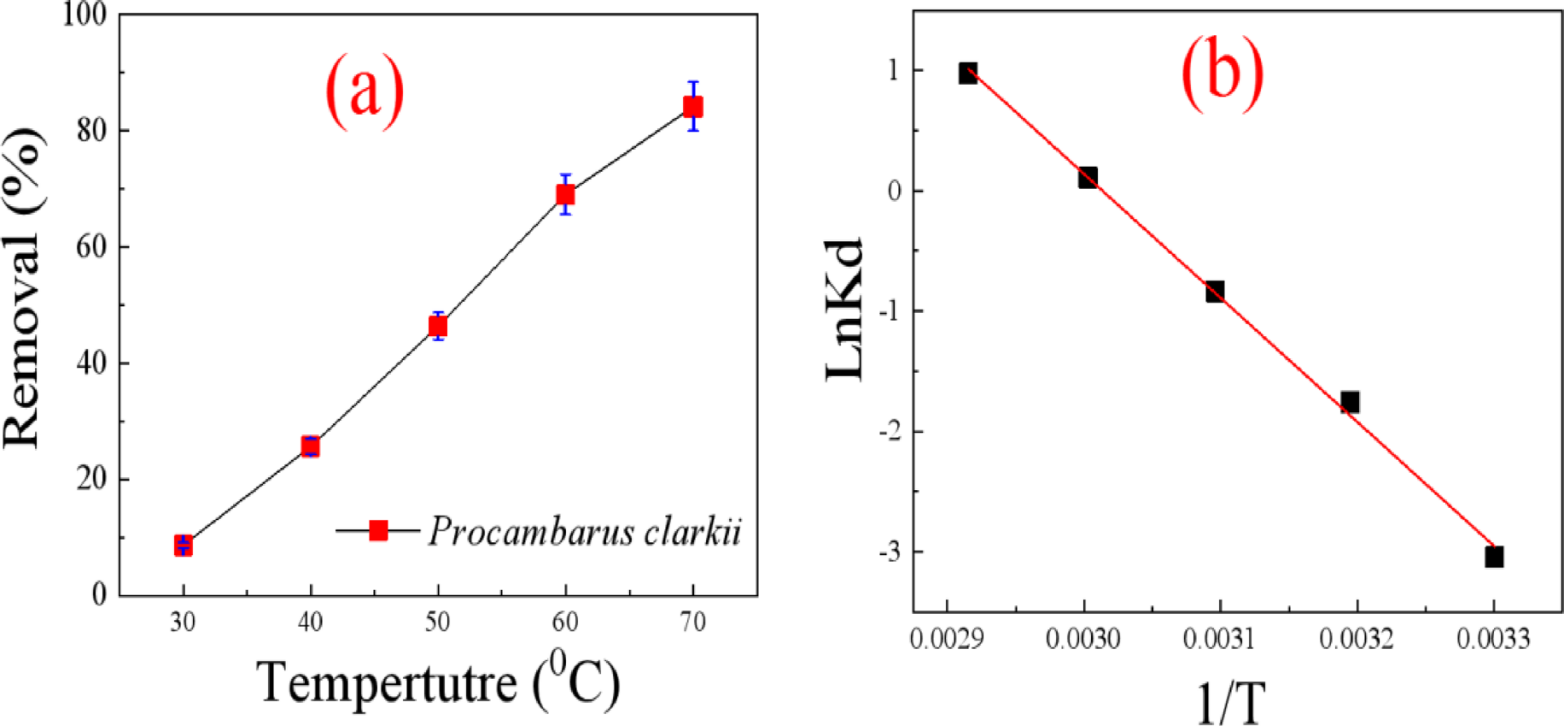
Effect of (**a**) temperature on removal of MR dye. (**b**) Van’t Hoff plots of MR adsorption onto the *Procambarus clarkii*.

Temperature is a significant parameter in the adsorption process because it affects immensely both the adsorption capacity and rate. To find the optimum temperature at which MR dye can be adsorbed onto *Procambarus clarkii*, batch experiments were carried out under a temperature range of 30 °C to 70 °C. For each experiment, 0.1 g of the adsorbent was measured into 50 mL of MR dye solution with an initial concentration of 25 mg/L and pH 7 and shaken under the same conditions until equilibrium. The objective was to study the effect of temperature on the adsorption efficiency and determine the best thermal conditions for maximum dye removal. As shown in Fig. 10a, in which the temperature was increased from 30 °C to 70 °C led to enhancement of MR dye adsorption efficiency, confirming that adsorption is an endothermic process^33^.

The thermodynamic parameters were determined to understand the adsorption behaviors. The equilibrium adsorption experiments were conducted at five distinct temperatures within a range of 25–70 °C. The change in Gibbs free energy ($\varDelta{\mathrm{G}}^{\mathrm{o}}$), enthalpy ($\varDelta{\mathrm{H}}^{\mathrm{o}}$), and entropy (${\varDelta\mathrm{S}}^{^\circ}$) were estimated using Eqs. 7–9^33^.

7 $$\ln K_{d} = \frac{{\Delta S^{^\circ } }}{R} - \frac{{\Delta H^{^\circ } }}{{RT}}~$$

8 $$\Delta G^{0} = \Delta H^{^\circ } - T\Delta S^{^\circ } ~$$

9 $$\Delta G^{0} = - RT\ln K_{{d~}} ~$$

Where ΔS° (J/mol. K), ΔG° (kJ/mol), and ΔH° (kJ/mol) represent the changes in entropy, Gibbs free energy, and enthalpy, respectively. T is the adsorption temperature, R is the gas constant.

As presented in Fig. 10b and the data in Table 3, the negative ΔH° value indicates that the adsorption process is exothermic, meaning that heat is released during adsorption. This implies that strong interactions exist between MR molecules and the functional groups on the carapace surface. The relatively high magnitude of ΔH° suggests that adsorption involves strong binding forces, which may include electrostatic attraction and surface complexation. The positive ΔS° value (0.259 kJ/mol·K) indicates an increase in randomness at the solid-solution interface during adsorption. This can be attributed to the displacement of water molecules from the biosorbent surface by MR molecules, resulting in greater disorder in the system. The ΔG° values are negative at all studied temperatures, indicating that the adsorption of MR is spontaneous^34^.

**Table 3:** Thermodynamic parameters for MR adsorption by *Procambarus clarkii*.

Adsorbent	T (K)	ΔG° kJ/mol	ΔH° kJ/mol	ΔS° KJ/mol. K
Procambarus clarkii	303	-0.164	-85.81	0.259
	313	-0.167		
	323	-0.169		
	333	-0.172		
	343	-0.175		

### Regeneration

**Fig. 11:**
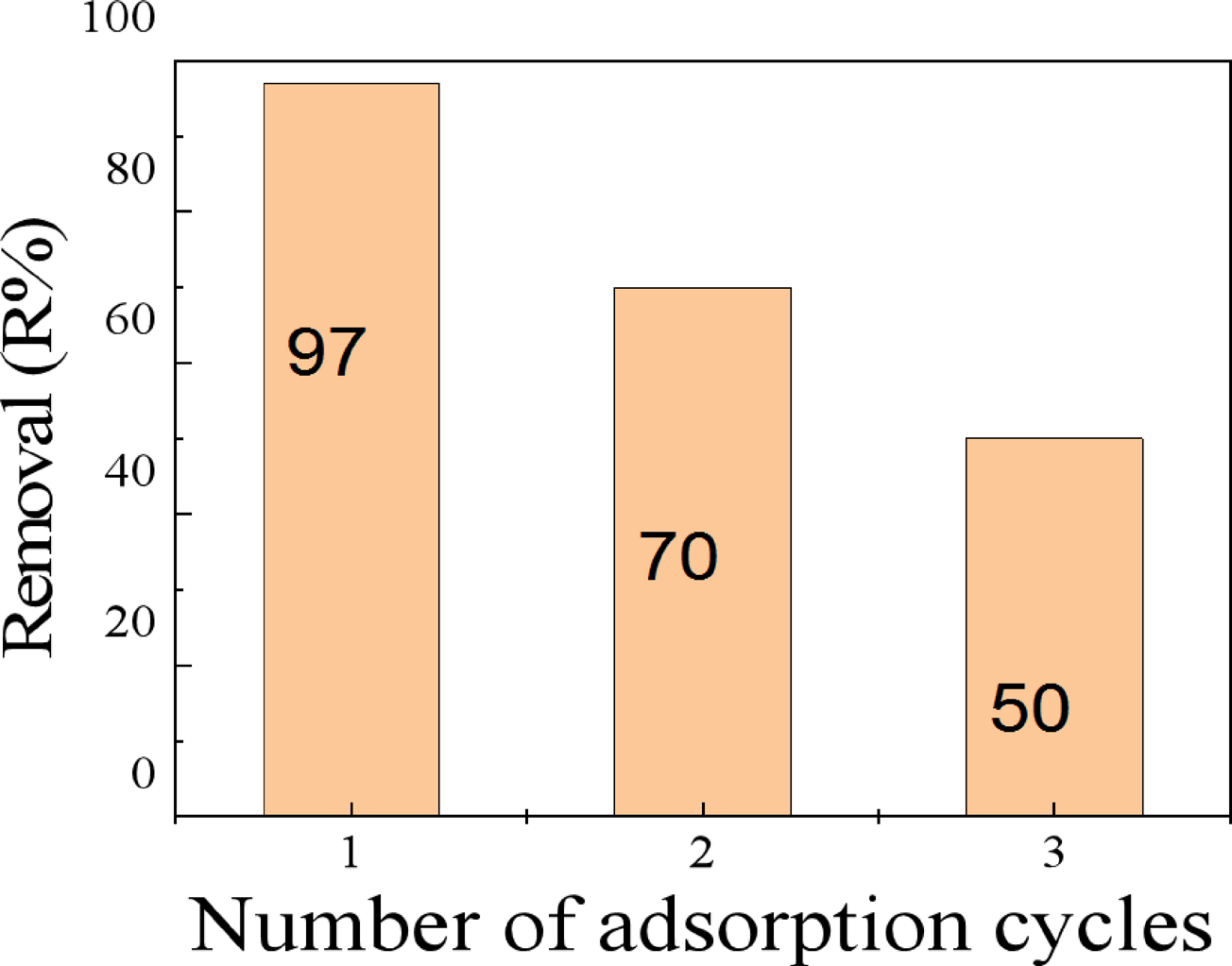
Regeneration of the *Procambarus clarkii* up to three successive desorption-adsorption cycles.

Performance of the Biosorbent in Reusability and Regeneration Cycles. The regeneration performance of the *Procambarus clarkii* carapace biosorbent is presented in Fig. 11, where successive adsorption-desorption cycles were carried out. The initial adsorption efficiency of the biosorbent, which is around 97%, indicates that the biosorbent has a high affinity for the methyl red dye. The adsorption efficiency of the biosorbent decreased to 70% during the second adsorption-desorption cycle, whereas it decreased to 50% during the third adsorption-desorption cycle. The gradual reduction of adsorption efficiency might be attributed to the blockage of some adsorption sites, structural fatigue of the biosorbent, and poor desorption of dye molecules from the biosorbent surface. The biosorbent, however, retained a considerable amount of adsorption efficiency even after successive adsorption-desorption cycles, indicating that the biosorbent is structurally stable. Procambarus clarkii carapace biosorbent, therefore, shows moderate reusability, which is acceptable for biosorbents. The regeneration performance of the biosorbent can be improved by optimizing regeneration conditions.

### Proposed adsorption mechanism of MR on *Procambarus clarkii* carapace

The adsorption of MR dye by the raw carapace of *Procambarus clarkii* can be attributed mainly to the synergistic effect of surface functional groups, electrostatic attraction, hydrogen bonding, and ion exchange, as facilitated by its hierarchical microstructure. The results obtained from zeta potential analysis revealed that the surface of the carapace has a positive charge at a pH of 7, as indicated by a zeta potential value of + 17 mV. The electrostatic attraction of anionic methyl red dye onto the carapace surface can be attributed to its positive charge at a pH close to or less than its point of zero charge, at which amino (-NH_2_) and hydroxyl (-OH) groups are protonated, enabling them to interact with negatively charged sulfonate groups in MR dye. The results obtained from FTIR analysis revealed that the carapace surface contains active functional groups such as -OH, -NH_2_, and carbonyl groups, which can form hydrogen bonds with electron-rich regions in methyl red dye. The presence of a high percentage of calcium carbonate in the carapace, approximately 40%, enabled ion exchange interactions between the carapace surface and methyl red dye, as revealed by XRD analysis.The protein and chitin-based organic matrix possesses a large number of binding sites that are capable of surface complexation, which further enhances the adsorptive affinity of the adsorbent. From the SEM and TEM images, it can be clearly seen that the adsorbent possesses a hierarchical, multi-scale porous structure that includes lamellar sheets, fibrillar bundles, and core-shell nanostructures. This enhances the adsorbent’s surface roughness and provides a large number of adsorption sites, which are beneficial for the rapid adsorption of the dye molecules. Although the BET surface area of the adsorbent is relatively moderate at 19.13 m²/g, the large number of active sites enhances the adsorption efficiency. From the kinetic plots, it can be clearly seen that the adsorption of the MR dye follows a pseudo-second-order kinetic model. This indicates that the adsorption of the dye molecules occurs in a step-wise manner: a rapid adsorption step due to electrostatic and hydrogen bonding forces, followed by a gradual intra-particle diffusion step, and finally an equilibrium step where the active sites are fully occupied and repulsive forces prevent further adsorption.

### Comparative adsorption of MR on various biosorbents

The maximum adsorption capacity of the *Procambarus clarkii* carapace for the removal of methyl red dye was found to be 14.39 mg/g, which is less than the maximum adsorption capacity of various chemically modified or engineered biosorbents as presnted in Table 4. Nevertheless, the fact that the carapace is unmodified and unactivated, unlike the aforementioned materials, is advantageous, as it is cost-effective and eco-friendly. Although the maximum adsorption capacity is moderate, the effectiveness of the biosorbent is evident, as it is able to remove up to 97% of the dye in just 2 h at a neutral pH, thus emphasizing the significance of surface functional groups and the hierarchical structure of the adsorbent on the effectiveness of the adsorption process. Furthermore, the fact that the invasive species is utilized as a biosorbent is advantageous, thus emphasizing the eco-friendliness and cost-effectiveness of the P. clarkii carapace, thus emphasizing its applicability to wastewater treatment technologies.

**Table 4:** Comparison of adsorption MR on various biosorbents.

Adsorbent	Adsorption Capacity (qm/, mg/g)	Ref.
*Procambarus clarkii* carapace	14.39	This work
Bali cow bone hydrochar	7.2	^35^
Rumex abyssinicus biochar	45.8	^36^
Charred Sal sawdust	70	^37^
Xanthated Sal sawdust	130	^37^
ZnO@Activated Carbon composite	45.99	^38^

## Conclusion

The present investigation shows the potential for the valorization of *Procambarus clarkii* carapace as a sustainable biosorbent material for the removal of methyl red from an aqueous solution. The adsorption capacity, irrespective of the low surface area, was dominated by the availability of chemically active groups rather than textural effects. Kinetic and diffusion analyses indicated a multistep adsorption mechanism dominated by fast surface adsorption and then intra-particle diffusion, while thermodynamic assessment showed the process to be spontaneous and exothermic in nature. Structural assessment of the material prior to and following adsorption confirmed its stability and thus its reliability for use. Although the adsorption capacity is moderate compared to chemically modified adsorbents, it has some advantages in terms of cost, eco-friendliness, and waste valorization. The regeneration study has proved that it has moderate reusability, where efficiency decreases with each cycle. This suggests that optimization of regeneration could improve its efficiency. This study has proved that *Procambarus clarkii* carapace has potential in wastewater treatment in a sustainable manner, in terms of eco-friendliness, and in waste management in a biological manner.

Future studies are recommended to investigate the adsorption capacity of the adsorbent toward other classes of contaminants, including cationic dyes, heavy metals, and emerging contaminants, also needs to be explored to extend the potential applications of the adsorbent. Finally, the use of this adsorbent in a continuous flow system and a pilot-scale test using real industrial wastewater needs to be explored to assess the practical viability of the adsorbent.

## Supplementary Information

Below is the link to the electronic supplementary material.

Supplementary Material 1

## Data Availability

The authors confirm that the data supporting the findings of this study are available within the article.
