# Supplementary material for: Transforming waste into worth: Procambarus clarkii carapace as a high-performance biosorbent for methyl red dye
Source: Sci Rep. 2026 Apr 2;16:11366. doi: 10.1038/s41598-026-44037-y (PMC13049000; doi:10.1038/s41598-026-44037-y)
Supplement: Supplementary file 1 — Supplementary Material 1 [file 41598_2026_44037_MOESM1_ESM.docx]

**Transforming Waste into Worth: Procambarus clarkii Carapace as a High-Performance Biosorbent for Methyl Red Dye**

Rofaida. F.H. Darweesh^a^, Abdelaal S.A. Ahmed^b*^, Remon M. Zaki^a^, Aldoshy Mahdy^c^

^a^ Environment Science Department, Faculty of Sugar and Integrated Industries Technology, 71516, Assiut University, Egypt

^b^ Chemistry Department, Faculty of Science, Al-Azhar University, Assiut 71524, Egypt.

^c^ Zoology Department, Faculty of Science, Al-Azhar University, Assiut 71524, Egypt.

**Corresponding author:** [abdelaalsaiyd@gmail.com](mailto:abdelaalsaiyd@gmail.com); [abdelaalsaiyd@azhar.edu.eg](mailto:abdelaalsaiyd@azhar.edu.eg) (Abdelaal S.A. Ahmed).

## Isotherm study

he Langmuir isotherm equation can be expressed mathematically as given in **Eq.3**.^24^

$$\frac{C{}_{e}}{q_{e}} = \frac{C_{e}}{q_{m}} + \frac{1}{\begin{aligned} K_{L} q_{m} \\ \end{aligned}} (Eq.3)$$

Where C_e_ (mg/L) is an equilibrium concentration, q_e_ (mg/g) is the adsorption capacity at equilibrium, q_m_ (mg/g) is the maximum adsorbent capacity, C_0_ (mg/L) is the initial concentration of MR dye, and K_L_ (L/mg) is Langmuir constant.

Freundlich adsorption isotherm is a semi-empirical equation for adsorption on inhomogeneous surfaces. It postulates that the amount adsorbed varies as a function of its equilibrium concentration in a non-linear fashion, suggesting an exponential distribution of adsorption sites with different energy levels. The model suggests that adsorption occurs by multilayer formation on surfaces of different affinities towards the adsorbate. The Freundlich isotherm equation represents how the equilibrium adsorption capacity and the equilibrium solute concentration are correlated, as illustrated in **Eq.4**.

$$\mathrm{Log}q_{e} = logK_{f} + \frac{1}{n}\log C_{e} (Eq.4)$$

Where, n is the adsorption intensity which should have a value between 0.10 and 1 for favorable adsorption. K_f_ is a Freundlich constant.

From the isotherm plots are shown on **Fig.S1** and the estimated parameters are categorized in **Table S1,** the higher R^2^ value suggests that the Langmuir isotherm model best describes the experimental data. This implies that a surface with uniform coverage of localized adsorption sites has produced a dye monolayer.^25^ The interaction between an absorbent and an adsorbate is typically described by the Langmuir constant K_L_. The estimated K_L_ value for MR adsorption by *Procambarus clarkii* is 2.40 L/mg as shown in **Table S1**. The maximum adsorption capacities of MR onto Procambarus clarkii were estimated to be 14.39 mg/g.


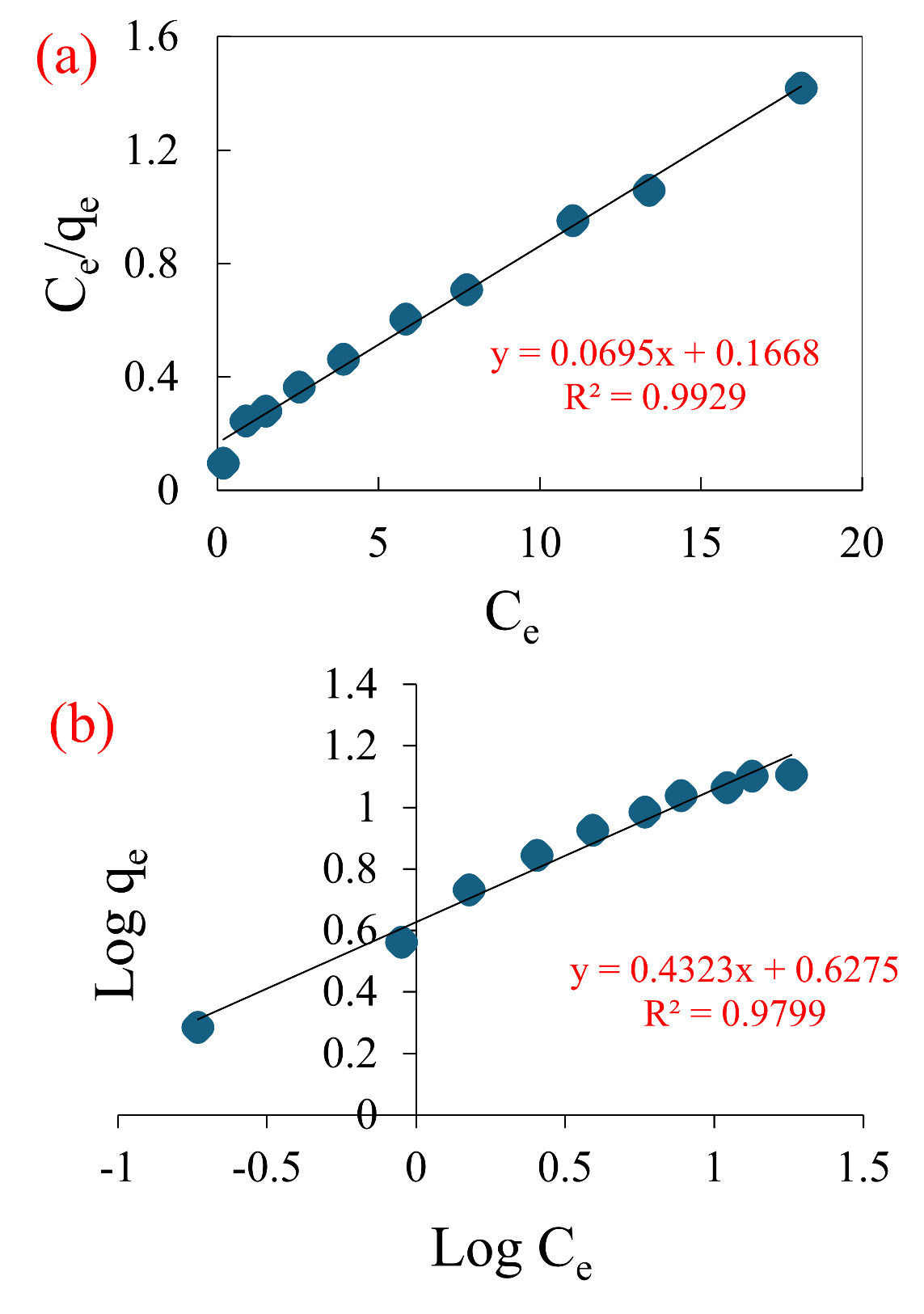


**Fig. S1**: Isotherm plots for the adsorption of MR dye on *Procambarus clarkii* carapace. (a) Langmuir and (b) Freundlich isotherm models.

**Table S1**: Parameters of Langmuir and Freundlich isotherm models for adsorption of MR on *Procambarus clarkii* carapace.

| Adsorbent | Langmuir model | | | Freundlich model | | |
| --- | --- | --- | --- | --- | --- | --- |
|  | K_L_  (L/mg) | q_m_  (mg/g) | R^2^ | K_f_  (mg/g) | N | R^2^ |
| *Procambarus clarkii* | 2.40 | 14.39 | 0.99 | 2.71 | 0.63 | 0.97 |

### 1-Adsorption kinetics

Adsorption kinetic experiments were conducted to get a better understanding of the mechanism and adsorption rate. Two linear kinetic models, the pseudo-first-order model and the pseudo-second-order model, were employed to describe the adsorption behavior of MR dye onto *Procambarus* *clarkii*. The pseudo-first-order model is described by **Eq. 5**, while the pseudo-second-order model is expressed by **Eq. 6**.^31^

$$\mathrm{Ln}\left( q_{e}-q_{t} \right)=Ln q_{e}-K_{1}t (Eq.5)$$

$$\frac{t}{q_{t}}=\frac{t}{q_{e}}+\frac{1}{k_{2}qe^{2}} (Eq.6)$$

Where k_1_ (min^-1^) and k_2_ (mg/g. min) are the pseudo-first and pseudo-second-order kinetic model, respectively.

From the fitted plots are represented in **Fig.S1**, and their related parameters are in **Table 1**, the adsorption of MR dye onto *Procambarus clarkii* obey the pseudo-second-order kinetic model, as evidenced by R² values. Furthermore, the observed q_e_ values calculated using the pseudo-second-order kinetic mode are close to the experimental values.


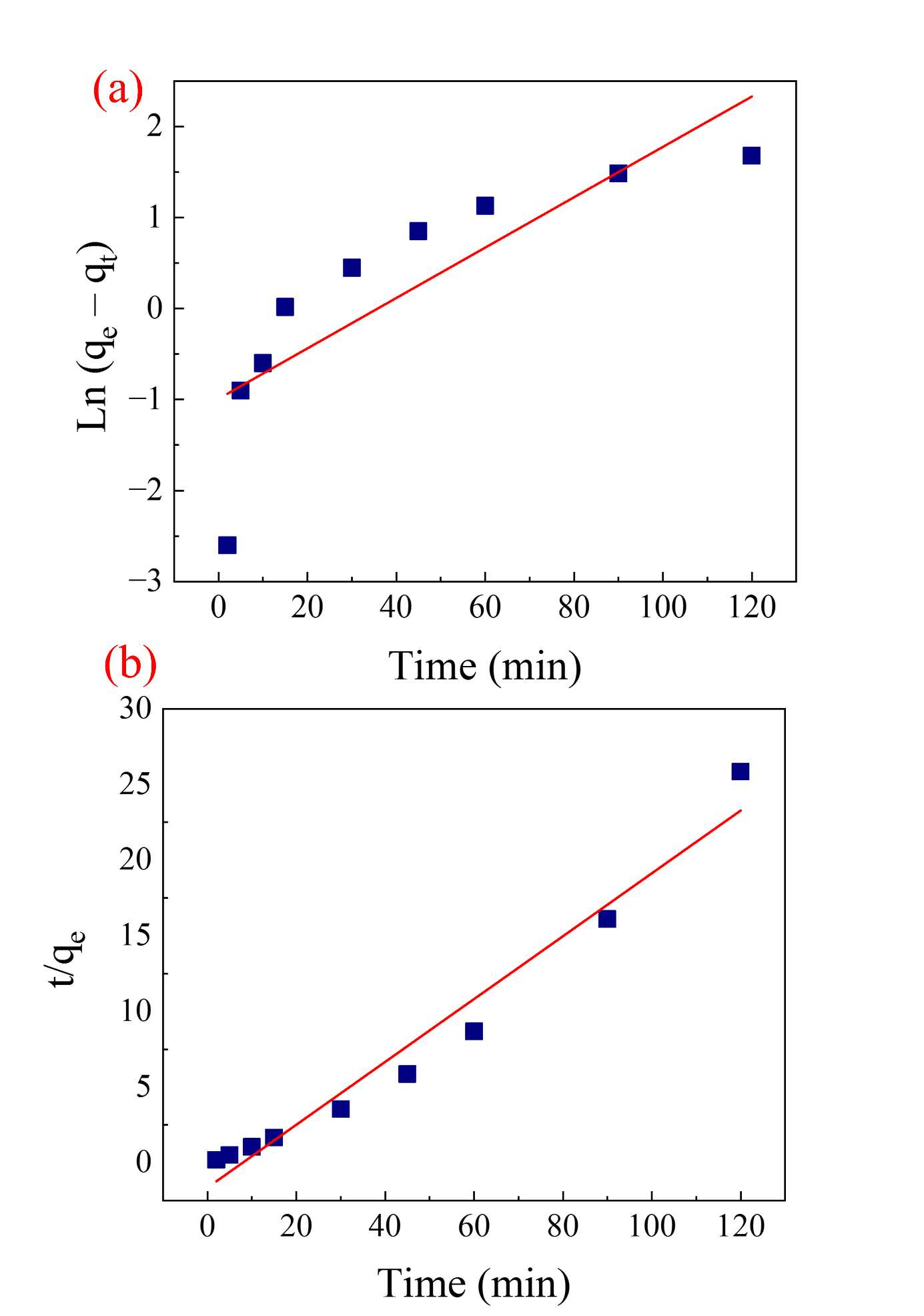


**Fig. S1**:(a) The pseudo-first order and (b) The pseudo-second order model of *Procambarus clarkii* carapace

**Table S1**: Parameters of the pseudo-first and pseudo-second order kinetic models for adsorption of MR on *Procambarus clarkii*.

| Adsorbent | Pseudo first order | | | Pseudo second order | | |
| --- | --- | --- | --- | --- | --- | --- |
|  | q_e_  (mg/g) | K_1_  (1/min) | R^2^ | q_e_  (mg/g) | K_2_ | R^2^ |
| *Procambarus clarkii* | 0.37 | 0.0002 | 0.8 | 4.82 | -0.03 | 0.96 |
